# Criteria pharmacists use to refer patients to a post discharge pharmacist review clinic

**DOI:** 10.1016/j.rcsop.2025.100647

**Published:** 2025-08-20

**Authors:** Adrienne Kostellar, Michael Barras, Ian Coombes, Andrew Hale, Carla Scuderi, Neil Cottrell, Nazanin Falconer

**Affiliations:** a Pharmacy Department, Royal Brisbane and Women's Hospital, Queensland Health, Cnr Butterfield Street and Bowen Bridge Road, Herston, QLD, Australia 4029; b School of Pharmacy and Pharmaceutical Sciences, The University of Queensland, UQ Dutton Park, Level 4, 20 Cornwall Street, Woolloongabba, QLD 4102, Australia; c Princess Alexandra Hospital, Queensland Health, 199 Ipswich Road, Woolloongabba, QLD 4102, Australia; d Discipline of Pharmacy, School of Clinical Sciences, Queensland University of Technology (QUT), Gardens Point Campus, 2 George Greet, Brisbane, QLD 4000, Australia

**Keywords:** Transition of care, Hospital pharmacy, Risk factors, Risk criteria, Medication related harm, Post discharge review

## Abstract

**Background:**

The transition from hospital discharge to primary care is a critical period in a patient's healthcare journey. Health system errors occur, due to a breakdown in communication or lack of structured planning which can lead to medication related harm or hospital readmission. At a quaternary referral hospital in Australia, pharmacists refer Internal Medicine patients to a pharmacist-led clinic for post-discharge medication review. While clinical resources exist to guide identification of at-risk patients, it remains unclear if and to what extent, pharmacists incorporate these criteria into their referral.

**Aim:**

To determine the criteria and reasons used by pharmacists to refer Internal Medicine patients to a post discharge pharmacist review clinic.

**Methods:**

Semi-structured interviews were conducted with hospital pharmacists who had worked in Internal Medicine and previously referred patients to the post discharge review clinic. Interviews were conducted until data saturation was obtained. Interviews were audio recorded, transcribed and coded using NVivo®. Themes and subthemes were identified through inductive thematic analysis and finalised via discussion within the research team.

**Results:**

Eleven pharmacists were interviewed. Five themes emerged describing referral criteria and reasons: (1) medication criteria including the use of high-risk medications and adjustments; (2) patient criteria including health status, frailty and social aspects of health including carer supports; (3) system pressures including patient flow and time constraints in care delivery; (4) post-discharge care including medication liaison and evaluation of tolerability and; (5) clinical judgement described as “worry” about the patient, highlighting the role of clinical reasoning.

**Conclusion:**

Pharmacists used established criteria from clinical resources to identify high-risk patients for referral; however, they also relied on clinical judgement. Referrals aimed to prevent medication related harm and improve communication with patients and healthcare providers. Future research should evaluate the effectiveness of clinical judgement to ensure high-risk patients are identified for transition of care services.

## 1 Introduction

The transition from hospital discharge to primary care is a critical period in a patient's healthcare journey. This period poses a heightened risk of communication errors and harm resulting from a disconnect in the continuity of quality use of medicines.^1^ Medications are essential in the management of many health conditions; however, their use is not without risk. Medication related harm (MRH) is estimated to contribute to over 250,000 Australian hospital admissions each year.^2^ Harm may be due to adverse medication reaction or event, medication error or medication related problems.^3^ Multiple factors increase the risk of MRH including polypharmacy,^4^ specific classes of medicines (for example, opioid analgesia or cardiovascular agents),^5^ older age,^6^ frailty,^6^ and medication non-adherence.^5^ Preventing MRH is a World Health Organisation (WHO) global priority particularly at transitions of care (ToC).^7^

Pharmacists play a crucial role in preventing MRH. Identifying patients who are at risk of MRH is critical to direct the delivery of ToC services in the transition from hospital to primary care. To do this, there are numerous checklists to assist pharmacists to identify at-risk patients. Typical checklists contain criteria such as risk factors for MRH,^8^ identifying medications to mitigate inappropriate medication prescribing to reduce MRH,^9^^,^^10^ or patient groups at-risk of MRH during ToC.^8^ Pharmacist activity in post discharge clinics is variable but can involve a medication history, medication reconciliation, and the identification and resolution of medication related problems in collaboration with medical teams. Post discharge review, particularly collaborative clinics, have demonstrated a reduction in 30-day hospital readmissions.^11^ Risk of MRH varies among patients so ensuring those patients who are at the highest risk are identified and referred can ensure efficient allocation of limited pharmacy resources.

In today's complex and busy healthcare environment, the practical application of clinical checklists and resources to systematically identify who is at risk, can be labour intensive as many contain an extensive list of criteria. Qualitative research exploring how pharmacists assess MRH risk at discharge remains limited. Prior studies have relied on multidisciplinary team opinions^12^^,^^13^ or consensus-driven survey methodologies,^14^ offering limited insight into the underlying rationale for selected criteria and limited pharmacist perspectives.^13^^,^^14^ It remains unclear if, and to what extent, hospital pharmacists incorporate checklist criteria into referral decisions for post discharge review.

A quaternary teaching hospital in Queensland, Australia, operates a post discharge pharmacist review clinic for all adult patients discharging from the Internal Medicine department who are returning to their own home. Approximately 10 % of discharged patients are referred to the clinic. Patients are referred by the Internal Medicine team pharmacist who completes a paper-based referral form (Supplementary material A). This form contains broad criteria known to influence MRH for the pharmacist to indicate referral reasons. The decision to refer relies on their clinical judgement. While these criteria help guide referrals, little is known about the criteria pharmacists select in practice. This study seeks to address this gap in knowledge.

## 2 Aim

To investigate the criteria pharmacists consider when referring a patient to a post discharge review clinic.

### 2.1 Objectives

1. To identify which criteria pharmacists consider when referring patients to a post discharge review clinic
2. To explore why those criteria are chosen when triaging patients for referral

## 3 Methods

### 3.1 Study design

This study used semi-structured interviews with hospital pharmacists. Ethics approval for this research was granted by the Hospital's Human Research Ethics Committee (HREC/2021/QRBW/75233). The consolidated criteria for reporting qualitative studies (COREQ)^15^ was adhered to throughout data collection and analysis (Supplementary material B).

### 3.2 Setting

This study took place at a 1000-bed quaternary referral hospital located in Queensland, Australia. The hospital pharmacy department employs approximately 120 pharmacists. Ten pharmacists provide clinical services for patients admitted to Internal Medicine. Inpatients receive a pharmacist admission medication history and reconciliation, clinical review, a medication list and education about changes to and ongoing medications at discharge. A senior pharmacist delivers the post discharge medication review clinic for half a day Monday to Friday. Patients are reviewed an average of three weeks after discharge.

### 3.3 Participants and recruitment

All eligible pharmacists with a Bachelor of Pharmacy degree, a minimum 12-months clinical experience, actively working in the Internal Medicine team or had completed a rotation in the 12 months prior to the study period, were invited to participate. All pharmacists had prior experience using the paper-based referral tool in routine clinical practice before the study period. Pharmacists were invited to respond to an expression of interest email to participate. Participation was voluntary and participants received no incentive to participate.

### 3.4 Interview structure

An interview guide was developed, informed by the literature and through discussion within the research team, focusing on areas known to increase the risk of MRH (Supplementary material C). This guided participants to provide in depth responses to the research objectives. During the interview, participants were invited to provide examples of criteria used in previous referrals. This allowed the participant to lead the interview. Practice interviews with pharmacists not eligible to participate were undertaken to pilot the interview guide, explore interview skills, and reflect on the interview process.

### 3.5 Data collection

The interviews were conducted by the research pharmacist (AK) between October 2022 to May 2023. AK was responsible for interview guide development, participant recruitment and consent. All participants provided written consent. Interviews were conducted face to face in a private setting to ensure confidentiality and were scheduled during department business hours at a time suiting the participant. All interviews were audio recorded with automated transcription by Microsoft Teams®. Following each interview, transcriptions were reviewed, and errors were manually corrected by AK. Participants did not review the transcripts. A reflective journal was kept, assisting with documenting interview observations and for reflection by the interviewer with regards to interesting discussion points which might inform themes for further investigation.

### 3.6 Data analysis

Transcriptions were reviewed after each interview to facilitate familiarisation of the data. All transcripts were deidentified and assigned participant numbers before undergoing analysis and coding using NVivo® software. Inductive thematic analysis, as described by Braun and Clarke,^15^ was employed. Transcripts were read multiple times to ensure thorough data immersion, with coding focused on information deemed relevant to clinic referral. Preliminary coding was independently applied for the first four transcripts by AK and subsequently cross-checked by an associate investigator (NC). O'Conner and Joffe recommend 10–25 % of data to be coded by two reviewers for intercoder reliability.^16^ After optimisation of coding, AK completed the coding of the remaining seven transcripts alone. Themes and subthemes were developed by AK and reviewed with NC using descriptions and illustrative quotes. The analysis was presented to the investigating team (NC, IC, AH, MB) for further discussion, leading to consensus on the final analysis.

### 3.7 Demonstrating rigor of results

To ensure research rigor, credibility, transferability, dependability and confirmability were addressed.^17^^,^^18^ The research team comprised of hospital pharmacists and university researchers with expertise in MRH, ToC, clinical pharmacy workflows and qualitative research methods. Clinical pharmacists with a range of years of experience were sampled.

The research pharmacist (AK) with 12 years of hospital pharmacy experience, conducted interviews, data collation and analysis whilst working in the post discharge clinic. Several measures were taken to minimise interview bias. Firstly, an interview guide developed by a wider research team enabled participants to provide examples of previous referrals. Secondly, AK maintained a reflective journal documenting learnings and improvements for future interviews. As participating pharmacists were colleagues to AK with no reporting hierarchy, they were encouraged to provide a true reflection of their experience to reduce positivity bias. Two research team members (IC and AH) held managerial roles within the department; to address the unequal relationship, pharmacists were advised their responses and transcripts would remain de-identified and confidential. To reduce bias in data analysis, regular feedback with the research team was undertaken and coding of interview transcripts was regularly reviewed with the research team to reach consensus.

## 4 Results

A total of 24 pharmacists were emailed an expression of interest with 13 respondents. Two respondents did not meet the inclusion criteria leading to the completion of 11 interviews. Data saturation was achieved with no new themes emerging from the data at interview 10. The average interview duration was 51 min. A spectrum of clinical experience was represented in the demographic profile of interviewees (45 % with 0 to 5 years registered practice and 54 % greater than 5 years registered practice).

Using inductive thematic analysis five themes were identified: (1) medication criteria, (2) patient criteria, (3) systems and pressures, (4) post discharge care and (5) clinical judgement. A total of 14 subthemes were created representing referral criteria (Table 1).

**Table 1:** Themes and subthemes categorised as criteria for patient referral.

Theme	Subtheme	Description
Medication criteria	High-risk and perceived high-risk medication	• APINCH medications • Perceived high-risk medication (e.g. anti-hypertensives, anti-epileptics, oral hypoglycaemics) due to risk of adverse effects
	Patient centric polypharmacy	• Polypharmacy (>5 medicines) influenced by medication and patient criteria (e.g. high-risk medications, ability to manage medications, patient health status and cognitive impairment)
	Medication changes and availability	• New medication(s), dose, frequency or formulation changes • Availability of patients own medications in hospital
	Medication management	• Aspects influencing physical management of medication (e.g. dexterity, vision impairment) • Intentional or non-intentional medication non-adherence
Patient criteria	Health status	• Change in health status (e.g. new disease diagnosis) requiring medication education • Referral of younger patients to foster positive healthcare engagement
	Frailty	• Patient factors influencing frailty (e.g. older age, falls, confusion or cognitive impairment) and impacts on safe medication management
	Social criteria	• Low health literacy and healthcare engagement • Limited formal or informal carer support systems • Limited ability to access healthcare
Systems and pressures	Patient flow	• Pharmacist time and environment pressures to complete medication counselling • Patients receiving large volume of information at discharge
	Staffing ratios	• Reduced weekend staffing ratios increasing time pressure for pharmacists
Post discharge care	Post discharge medication review	• Medications changes enacted post discharge, follow up monitoring completed, and medications reviewed by primary care providers
	Patient outcomes	• Post discharge review of medication tolerability and disease state management
	Post discharge liaison	• Communication with primary care providers and barriers to ToC communication • Communication with support network
Clinical judgement	Tacit knowledge	• Impact of clinical experience on referral, feeling “worried”
	Patient centric referral	• Individualised referral based upon patient and medication criteria described above

Key: APINCH: A: antimicrobials, P: potassium and electrolytes, I: insulin, N: narcotics and sedatives, C: chemotherapeutic agents, H: heparin and anticoagulants. ToC: Transition of Care.

### 4.1 Theme One: Medication criteria

Pharmacists used medication criteria from clinical checklists^8^, ^9^, ^10^ to inform their referral decision. Particular attention was given to high-risk medications especially those adjusted in hospital, and aspects influencing safety of medication use following discharge.

#### 4.1.1 High-risk and perceived high-risk medications

Use of high-risk medications was a common referral reason. This was denoted by the APINCH acronym used within Australian acute healthcare settings to promote the awareness of medications with a high safety risk (A: antimicrobials, P: potassium and electrolytes, I: insulin, N: narcotics and sedatives, C: chemotherapeutic agents, H: heparin and anticoagulants).^19^ In addition to this category, pharmacists referred patients taking cardiac (including antihypertensive and antiplatelet therapies) and oral hypoglycaemic agents as these are known to have a high risk of MRH or poor health outcomes secondary to non-adherence.^20^

*[P11]: “I would put more focus on the higher risk sort of medications, it's got a narrower therapeutic index… warfarin …. someone that's on a NOAC…”*.

*[P1]: “…anything that changes their blood pressure … falls can happen from that.”*

#### 4.1.2 Patient centric polypharmacy

Pharmacists considered polypharmacy (five or more medications) as a factor for referral. However, the total number of medications was not seen as problematic for all patients and this was assessed in relation to the patient's ability to manage their medications, comorbidities, cognitive function, and the types of medications prescribed.

*[P4]: “we see lists of 10, 15, 20 medications and can be very overwhelming for the patients. Sometimes it's not. Sometimes they're very aware of what the medications do and the roles they play and how important they are.”*

#### 4.1.3 Medication changes and availability

Pharmacists referred patients when medications changed due to a new diagnosis, to optimise disease management or due to changes in their renal function. Increasing complexity of specific dosing schedules, administration or storage requirements (e.g. refrigerated insulin) or combinations of high-risk medications were considered. Pharmacists considered patient cognition and social supports in the holistic perception of medication complexity.

*[P3]: “if the patient has gone from having no medications to a whole bunch of medications, and this might be…that first real significant illness in their life where they've had to deal with medications.”*

*[P6]: “For an older patient you know they might only be on a handful of medications but if it did include an anticoagulant and …a sleeping tablet and a blood pressure medication…It's probably not complex, but it's high-risk. …it would depend on the patient I guess in terms of the number of medications and…their ability to manage.”*

Referral became important when the patient did not bring their own medications to hospital, preventing pharmacists from removing discontinued medications or updating packaging to reflect revised dose, timing or frequency instructions.

*[P10]: “if we…change them from…one anticoagulant to another…And if they hadn't brought in their medicine…and I couldn't …physically take away that old one….”*

#### 4.1.4 Medication management

Pharmacists identified poor or variable adherence with a planned medication regimen and the patient's ability to self-manage or administer medications as aspects influencing safe and effective medication use following discharge. Pharmacists used clinic referral as an opportunity for the clinic pharmacist to reassess medication instructions, safety and level of adherence or to assess the impact of modifications in physical management of medicines (e.g. initiating a dose administration aid). Non-adherence was described by all pharmacists as a referral criterion, for patients with declining cognition, limited health literacy and healthcare engagement.

*[P9]: “…taking things sporadically or they're not taking certain things whether it's a memory thing that's making them forget….”*

*[P6]: “I'm worried that the patient may not be adherent either because there's a lot of information overload in hospital, they've never had to manage medications before….”*

Pharmacists evaluated factors affecting a patient's or carer's ability to manage medications, including functional ability, dexterity, and vision or hearing impairments.

*[P10]: “they're visually impaired as well or hard of hearing…if they can't see the list …how are they going to go home and confidently and safely know how to take their medications….”*

*[P4]: “say we've given you this device (to pack medications) that's supposed to fix everything, is it actually any easier for you to manage with the dose administration aid?.”*

### 4.2 Theme Two: Patient criteria

Pharmacists identified that health and demographic criteria, relating to the patient, raised their concerns about the patient's ability to manage their medicines safely after discharge.

#### 4.2.1 Health status

Pharmacists recognised a change in patient health status, particularly the diagnosis of a new chronic condition often resulted in medication changes and the potential for information overload. Referrals provided opportunity for further education, assessment of medication adherence and to facilitate continued engagement with primary healthcare for medication titration, review and monitoring. Referral of younger patients due to new disease diagnoses was often considered if the patient was taking high-risk medications or polypharmacy. The referral was viewed as an opportunity to foster positive healthcare engagement and reduce risk of long-term health complications.

*[P4]: “if someone had a new diagnosis of …for example heart failure that's five medications that just get thrown on immediately and…it's just overwhelming to suddenly have to take all these medications.”*

*[P9]: “new insulin where you've got young patients even in their teens and 20s …it's a bit of a lifestyle change …the more positive interactions they have in healthcare the better….”*

#### 4.2.2 Frailty

Frailty was described as a referral criterion by five pharmacists. Pharmacists described aspects associated with frailty such as older age, falls and cognitive impairment, indicating these characteristics are part of their referral criteria.

*[P4]: “…can't manage as well at home and whether that's physically they're struggling with dexterity…they just don't have the same capacity to process everything….”*

*[P4]: “probably 65 and above is when you're starting to think about maybe age having a component in their comorbidities or their functional status.”*

Most pharmacists cited memory concerns, with or without diagnosis of cognitive impairment or dementia. Discharging pharmacists subjectively assessed patient comprehension during medication counselling through non-verbal cues, verbal prompts or overt patient concern. Pharmacists were concerned about impact on adherence including missed medication doses, drug duplication and MRH secondary to high-risk medications. Referral was considered as an opportunity for a review of medication management post-discharge and to optimise medication support.

*[P7]: “cognitive impairment with older people…more at risk of falls….”*

*[P6]: “…if their cognition is declining and if they're socially isolated in their on high-risk medications.”*

*[P11]: “…our elderly patients in that they don't process information as fast as a younger person. And so everybody's coming at them with you know like a package full of information….”*

#### 4.2.3 Social criteria

Concerns regarding low health literacy and low healthcare engagement, limited support networks, such as formal carers, and limited access to primary care services were all identified by pharmacists as reasons for referral due to the risk of medication non-adherence.

*[P10]: “if they have lack of insight, lack of understanding, maybe like a poor health literacy and then nonadherence.”*

*[P1]: “only see their GP once every ten years…the chances of them going and being compliant at the time it probably less.”*

Pharmacists also described assessing the carers' ability to support the patient post discharge. Whilst some carers were identified as being readily available to the patient (e.g. a spouse or partner living in the same household), pharmacists felt referral was required if they perceived the carer as being overwhelmed or frail.

*[P1]: “…Like if it*'*s a husband or wife who is equally as frail…I am more likely to refer that group of people than someone, say a paid carer who… is able to take on what you*'*re saying and then actually action it.”*

### 4.3 Theme Three: Systems and pressures

This theme described the perceived barriers pharmacists faced to effective medication communication at discharge. Pharmacists referred to the clinic to further communicate medication plans to both the patient and primary care.

#### 4.3.1 Patient flow

Pressure to expedite patient flow with timely discharges limited pharmacists time to assess patients' understanding of medications and resolve potential medication related problems prior to discharge. Pharmacists felt the volume of information delivered by multidisciplinary staff at discharge could affect patients' retention of important points of medication education.

*[P7]: “…I get the feeling that they (patients) are either overwhelmed with the degree of information they're getting on discharge, that there's a lot of information they may not remember.”*

*[P8]: “sometimes you just have to get a patient out and you don*'*t necessarily have the time to be able to sort out their problems…”*.

#### 4.3.2 Staffing ratios

Reduced pharmacy department staff to patient ratios on weekends and the need to cover workloads throughout the week, meant some pharmacists were unfamiliar with the patient they were responsible to discharge. On weekends, pharmacists had difficulty documenting medication changes in the medication discharge summary when the rationale for changes was not explicitly written in the medical record. Therefore, pharmacists referred if they considered the patient required further medication review.

*[P5]: “where you*'*re discharging somebody on the weekend that been here for quite some time and there*'*s been lots of medication changes and they might not be completely clear”.*

*[P6]: “…trying to organise anything like a dose administration aid or additional assistance on the weekend is very difficult”.*

### 4.4 Theme Four: Post discharge care

This theme describes the reasons why pharmacists request follow-up by the clinic pharmacist once the patient returned to the community setting.

#### 4.4.1 Post discharge medication review

Post discharge review of medications was a highly cited criteria for referral. This included confirming patients enacted medication changes, engaging the patient and primary care to follow up blood tests and disease state monitoring, and providing advice on dose adjustment.

*[P7]: “make sure that they've actually stopped the medications that we've stopped and replaced them with the new ones, and they're not doubling up on things.”*

*[P5]: “…you reduce a dose…like rivaroxaban for example but once their kidneys are functioning a bit better the dose may need to be increased again.”*

#### 4.4.2 Patient outcomes

Pharmacists explained how brief hospital admissions and multiple medication changes limited their ability to assess the effect of new medications or dose modifications. Evaluating side effects, tolerability, and disease state management was important with particular concern for APINCH and cardiovascular medications during initiation or dose modification.

*[P11]: “to make sure that what we've done is… having the effect that it's meant to have…and not caused some unnecessary harm.”*

#### 4.4.3 Post discharge liaison

Pharmacists viewed the post discharge service as an important avenue to ensure medication plans were communicated to the GP. Pharmacists believed the service could provide support and advice for medication safety and optimisation. They also saw value in the post discharge pharmacist communicating with support networks for the patient.

*[P6]: “…the high-risk discharge service is in a good position to help to advocate to make sure that you know the patient does see their GP and then also that the GP has all of the information that they need to make the best decisions about those medication doses.”*

*[P4]: “if the patient doesn't have maybe the capacity to manage the medicines are they gonna be able to pass that information on to the person who does?.”*

### 4.5 Theme Five: Clinical judgement

Pharmacists described the use of clinical judgement to refer patients through the application of tacit knowledge and descriptions of patient centric referrals.

#### 4.5.1 Tacit knowledge

Pharmacists described situations where they referred because of feeling “worried” possibly reflecting tacit knowledge and prior clinical experience. When asked to describe the clinical reasoning for this decision pharmacists frequently linked this to a perceived lack of patient cognition and comprehension of information, when high-risk medications and non-adherence were accompanying criteria. Often the assessment of “worried” occurred at the patient bedside during medication education.

*[P11]: “Am I slightly worried? Am I really worried?… you do sort of I guess fall back on that….”*

*[P6]: “it could be a younger male who*'*s not taken medications before…, an older patient who I just don't think is going to manage their medications appropriately….a high-risk medication, …a new medication…changes to medications….”*

*[P2]: “it comes down to again that unknown feel when you're talking to the patient of them trying to get their head around it”*.

#### 4.5.2 Patient centred referral

Pharmacists reflected how the decision to refer a patient was multifaceted, influenced by individual patient needs. Rather than relying on referral criteria in isolation, they considered multiple factors including, medication complexity, patient-specific conditions and the importance of effective communication.

*[P1]: “treat the person not the number scenario.”*

*[P11]: “…it's patient. It's condition. And its medication and the impact of that medication on I guess the patient and the condition….”*

## 5 Discussion

This study used semi-structured interviews to identify the criteria Internal Medicine pharmacists use to refer patients to a post discharge pharmacist review clinic and explored why pharmacists selected these criteria. Five themes were identified from the analysis of the interviews, including (1) medication criteria, (2) patient criteria, (3) systems and pressures, (4) post discharge care, and (5) clinical judgement. Pharmacists described referrals as a mechanism to optimise medication management, to prevent MRH and to ensure effective communication with patients, caregivers and primary care providers. Pharmacists employed an individualised, patient centred approach with multiple criteria considered for each patient.

### 5.1 Criteria pharmacists use and the link to current risk tools and checklists

Pharmacists applied recognised medication and patient criteria proven to result in MRH including frailty, cognitive impairment, non-adherence, high-risk medications and medication changes. These criteria are identified in Australian clinical pharmacy standards,^8^^,^^21^ and specific medication criteria within clinical resources such as BEERS and STOPP/START tools.^9^^,^^10^ These factors align closely with findings from previous qualitative research, identifying polypharmacy, medication changes and high-risk medications as criteria for post-discharge review.^12^, ^13^, ^14^ Polypharmacy and living alone are also criteria identified within risk stratification algorithms.^22^^,^^23^ While the Australian APINCH framework guides identification of high-risk medications which is subsequently reflected across risk assessment tools,^8^^,^^19^^,^^21^ pharmacists identified cardiovascular agents as high risk in this population. Cardiovascular agents are a known cause of preventable post discharge MRH.^5^^,^^20^ Interestingly, while risk tools^8^, ^9^, ^10^ and algorithms^24^ focus on older adults, defined as over 65 years, pharmacists in our study also referred younger patients, irrespective of age barrier. The benefits of extending ToC services to a younger population has been demonstrated previously, with a 25 % reduction in hospital readmissions among patients aged 51–65 years following post discharge pharmacist review.^25^ In our study, pharmacists cited potential MRH associated with high-risk medications, medication non-adherence and future impact on chronic disease management as their reason for referring younger patients. Overall, this study highlights pharmacists regularly apply criteria found within clinical checklists and references to known risk factors for MRH.

### 5.2 Communication challenges at ToC

Improving medication communication with patients, care providers and primary care was important to pharmacists. System challenges was an identified reason impacting communication in this study. This included time constraints, reduced staffing ratios, and pressure to facilitate patient flow, which were perceived by pharmacists as significant communication barriers. Discharge counselling may be rushed due to the urgency to free hospital beds, leaving pharmacists concerned about overwhelming patients with information and their ability to understand medication changes. These issues have been previously reported by patients following counselling.^26^, ^27^, ^28^, ^29^ Hospital clinicians have similarly highlighted gaps in medication knowledge as risks for post discharge MRH, including accidental duplication of medications and dosing errors.^12^ Similar concerns were also echoed in this study, with pharmacists attempting to address this risk by referral to the clinic where the clinic pharmacist can reinforce vital medication information with the patient. In addition to verbal counselling, effective written communication between hospital and primary care providers is critical. While a pharmacist prepared discharge medication list is commonly found in Australian discharge summaries,^30^ there has been limited detail on the rationale for medication changes.^31^ The risks identified in our study such as covering colleagues' workloads, unfamiliarity with patients and incomplete documentation of medication changes at discharge, may be contributing factors to this gap in information. While ToC referral may help address these issues, it also highlights the continued need to document medication information throughout the course of the patient admission to ensure clear communication with patients and primary care.

### 5.3 Use of clinical judgement

This study did not assess if pharmacists systematically applied checklist criteria for referral in each case. They described a subjective ‘worried’ criterion, drawing upon tacit knowledge shaped by their clinical experience,^32^ and highlights the significance of experiential learning in making assessments beyond standardised clinical checklists. When asked to articulate their clinical reasoning, pharmacists referenced a combination of patient cognition, frailty, non-adherence and use of high-risk medicines. Prior research has shown that pharmacists often struggle to articulate processes involved in clinical decision making.^33^ Documenting and communicating their concerns is important at ToC to ensure patient safety, reduce errors and optimise care across healthcare teams.^34^

Although clinical judgement is important, its limitations in ToC have been demonstrated in a study of junior doctors, who were unable to predict patient MRH within eight weeks of discharge.^35^ To the best of our knowledge, there are no studies investigating pharmacists ability to subjectively predict patients at risk of MRH, representing an important opportunity for future research. The clinical judgement of pharmacists may be shaped by clinical training and shared learnings within the workplace.

### 5.4 Challenges of current system

The clinic described in this study uses a paper-based referral form with broad criteria to identify reasons for referral. Of practical concern is the time constrained working environment, limiting the efficiency of a paper-based referral system, which may result in missed referral of high-risk patients. Given these constraints, optimising patient identification is important to ensure effective use of clinical resources. Predictive risk models using automated statistical methods, offer an alternative approach to identify patients at risk of readmission^22^^,^^23^^,^^36^^,^^37^ or post-discharge MRH.^24^ While these models may be more efficient and mitigate the burden of manual assessment of criteria, their clinical use is still evolving. Statistical algorithms also fail to capture the nuances in patient-pharmacist interactions gained at the patient bedside which were described in this qualitative study. A hybrid approach, integrating clinical judgement with automated tools is likely needed for optimal prioritisation of high-risk patients.

### 5.5 Study limitations

This study investigated the opinions of 11 pharmacists working in Internal Medicine at a single hospital site, representing a small sample of the Australian hospital pharmacy workforce. Despite the sample size, data saturation was achieved with the 11 interviews. Pharmacists identified criteria for MRH believed to be important for an Internal Medicine population, it remains unclear if these criteria are applicable across other specialty fields, where differing comorbidities and medications may influence risk assessment. Whilst there was a spectrum of years of experience, the sample size meant it was difficult to establish if duration of clinical experience impacted decision making process which would be important to explore further.

## 6 Conclusion

This study highlights the importance pharmacists place on medication safety at ToC to reduce the risk of MRH and enhance patient safety. Pharmacists described a patient centric referral process acknowledging medication risks, patient characteristics and the impact of health system constraints. While pharmacists used established MRH criteria, this research highlighted pharmacists also relied on clinical judgement and tacit knowledge. However, this subjectivity presents a challenge to ensuring consistency in referral of all high-risk patients. Future research should explore the application of clinical judgement to determine risk of post discharge MRH.

## CRediT authorship contribution statement

**Adrienne Kostellar:** Writing – review & editing, Writing – original draft, Methodology, Data curation. **Michael Barras:** Writing – review & editing, Writing – original draft, Supervision, Methodology, Conceptualization. **Ian Coombes:** Writing – review & editing, Supervision, Methodology, Conceptualization. **Andrew Hale:** Writing – review & editing, Methodology, Conceptualization. **Carla Scuderi:** Writing – review & editing, Supervision. **Neil Cottrell:** Writing – review & editing, Supervision, Methodology, Conceptualization. **Nazanin Falconer:** Writing – review & editing, Supervision.

## Declaration of generative AI and AI-assisted technologies in the writing process

During the preparation of this work the author (AK) used Microsoft CoPilot® for grammatical suggestions. After using this tool/service, the author (AK) reviewed and edited the content as needed and takes full responsibility for the content of the published article.

## Funding sources

This research was completed with the University of Queensland Research Training Program Tuition Fee Offset.

## Declaration of competing interest

The authors declare that they have no known competing financial interests or personal relationships that could have appeared to influence the work reported in this paper.

## Appendix A Supplementary data

Supplementary material A Image 1

Supplementary material B Image 2

Supplementary material C Image 3
