# Supplementary material for: Criteria pharmacists use to refer patients to a post discharge pharmacist review clinic
Source: Explor Res Clin Soc Pharm. 2025 Aug 20;20:100647. doi: 10.1016/j.rcsop.2025.100647 (PMC12712588; doi:10.1016/j.rcsop.2025.100647)
Supplement: Supplementary file 1 — Supplementary material A [file mmc1.pdf]

Queensland Government

Royal Brisbane & Women's Hospital
High Risk Discharge Pharmacy Service

HIGH RISK DISCHARGE (HRD) PHARMACY FOLLOW UP REFERRAL TOOL

(Complete if applicable)

URN:

Family Name:

Given Names:

Address:

Date of Birth: Sex: ☐ M ☐ F ☐ I

Date of referral: ____/____/____ **Discharge date:** ____/____/____ Team: ____________

Pharmacist review on discharge: ☐ Yes ☐ No

Patient notified of HRD Pharmacy referral: ☐ Yes ☐ No Phone number: ____________

Medicine management:

Managed by: ☐ Self ☐ Family / Carer ☐ Other: ____________

Websterpack: ☐ Yes ☐ No Dosette box: ☐ Yes ☐ No Other: ____________

Reason for referral (select multiple if required):

☐ Suspected non-compliance
☐ Significant medication changes / additions during admission
☐ Reinforcement for medication counselling (define below)
☐ Difficulty managing medicines
☐ Medication requiring therapeutic monitoring (define below)
☐ Other: ____________

Discharge liaison:

☐ Correspondence to GP (e.g. letter)
☐ Medication list faxed to GP
☐ Community pharmacy
☐ RACF
☐ Other: ____________

Prescription Information:

☐ Script not required
☐ Script given to patient
☐ Script dispensed by hospital pharmacy
☐ Patient did not receive prescription
☐ Other: ____________

Reason for admission: ____________

Relevant previous history: ____________

Issues for follow up (e.g. bloods, restarting medication, counselling):

____

Other relevant information:

____

Requested time period for follow up (if not standard): ____________

Referrer details:

Name: ____________ Signature: ____________ Designation: ____________ Date ____/____/____

Outpatient Review Dates (HRD Pharmacist):

Review 1: ____/____/____ (7-10 days post discharge) Review 2: ____/____/____ (30 days post discharge)
