# Supplementary material for: Criteria pharmacists use to refer patients to a post discharge pharmacist review clinic
Source: Explor Res Clin Soc Pharm. 2025 Aug 20;20:100647. doi: 10.1016/j.rcsop.2025.100647 (PMC12712588; doi:10.1016/j.rcsop.2025.100647)
Supplement: Supplementary file 3 — Supplementary material C [file mmc3.docx]

**Semi-structured interview guide**

**Interview introduction:**

Thankyou for agreeing to participate in this research project. We hope this research will help us to improve the healthcare services that we offer at the RBWH.

**Structure of interview:**

Today we will be talking about the high-risk discharge clinic. The purpose of the research is to explore the opinions of pharmacists on why they choose to refer people to the high-risk discharge clinic. Today’s interview will start by asking you general questions about your experience as a pharmacist. We will then move on to explore your views on referring people to the clinic.

**Information about the process:**

Today’s interview will be recorded and your responses transcribed and the audio recording deleted afterwards. Your transcribed responses will be coded for anonymity so other members of the research team are not aware of who provided responses. If at any time you don’t wish to answer the question, please let me know. If at any time, you wish to stop the interview and exit the study, you may do so, please let me know. Please refrain from using patient names or names of other healthcare staff for confidentiality purposes. If you inadvertently do, this will not be transcribed.

There are no right or wrong answers to the questions, I am interested in your views and your experience in this area. I may write some brief notes, they are for me, so I don’t miss any important information you have said. Do you have any questions about any of this information? Are you happy to proceed with the interview?

**Experience as a pharmacist:**

1. **I would like to start today with you telling me about your experience as a pharmacist. Can you tell me about what your role is at the Royal Brisbane and Women’s Hospital and experience as a pharmacist?**
2. ***[prompt if required]* Can you tell me your years of practice as a general registered pharmacist (not including intern year)?**
   1. 1-2 years
   2. 3-4 years
   3. 5-10 years
   4. 10+ years
3. **I would also like to know about your qualifications for this research study. Can you tell me your highest university qualification?**
   1. Bachelor of Pharmacy
   2. Grad Certificate of Pharmacy
   3. Grad Diploma of Pharmacy
   4. Master of Pharmacy
   5. Higher degree by research
   6. Other: ______________

**Introductory phase: views and perceptions on who they usually refer to the high risk discharge clinic**

**I will now proceed to the next part of the interview. The high-risk discharge clinic accepts referral for general medicine patients for post discharge follow up review by a pharmacist. There is a referral form, with loose referral criteria but you can refer outside of this criteria.**

**Q. Can you start by generally describing the last time you referred patient(s) and can you explain to me why you chose to refer them to the high risk discharge clinic?**

*What has led you to consider these ideas / reasons to refer?*

*What factors influence you to refer some people over others?*

*Why do you consider some factors over others?*

*Do some factors have more “weight” than others?*

**Prompt questions if medicine factors are brought up:**

**You have mentioned some medication factors that influence referral [insert examples from participant]. Can you tell me more about this?**

*Can you give me an example of the medication factors that influenced your referral?*

*What are the reasons for choosing the factors you described to me?*

*Can you describe if these factors influence you to refer some people more than others? Why?*

**Prompt questions if patient factors are brought up:**

**You have mentioned some factors about the patient that influence referral [insert examples from participant]. Can you tell me more about this?**

*Can you give me an example?*

*What are the reasons for choosing the factors you described to me?*

*Can you describe if these factors influence you to refer some people over others? Why?*

**Prompts if environment factors are mentioned:**

**You have mentioned some external/environment factors that influence referral [insert examples from participant]. Can you elaborate more about this example or other times when this influenced your referral?**

*What are the reasons for choosing the factors you described to me?*

*Are there any other situations/examples where you referred for this reason?*

*How did this affect decision to refer?*

*Can you describe if these factors influence you to refer some people over others? Why?*

**Prompts if communication factors are mentioned:**

**You have mentioned some communication factors that influence referral [insert examples from participant]. Can you elaborate more about this example or other times when this influenced your referral?**

*Can you describe how communication affects your referrals? Situation? What happened? Tell me more about this.*

*Why/how does this influence your decision to refer?*

*Can you describe if these factors influence you to refer some people over others? Why?*

**Summarise interview:**

**So the factors we discussed today are (list out areas discussed). Was there anything else that I may have missed or you wished to discuss further that you brought up?**

**Conclusion:**

**This is all the questions I had to ask you and to talk about today. Do you have any final thoughts on our discussion today or anything that you would like to follow-up on? Thankyou for participating in this research.**
